# Bovine Ephemeral Fever in Asia: Recent Status and Research Gaps

**DOI:** 10.3390/v11050412

**Published:** 2019-05-03

**Authors:** Fan Lee

**Affiliations:** Epidemiology Division, Animal Health Research Institute; New Taipei City 25158, Taiwan; fanlee@mail.nvri.gov.tw; Tel.: +886-2-26212111

**Keywords:** Bovine ephemeral fever, *Culicoides* biting midge, mosquito

## Abstract

Bovine ephemeral fever is an arthropod-borne viral disease affecting mainly domestic cattle and water buffalo. The etiological agent of this disease is bovine ephemeral fever virus, a member of the genus *Ephemerovirus* within the family *Rhabdoviridae*. Bovine ephemeral fever causes economic losses by a sudden drop in milk production in dairy cattle and loss of condition in beef cattle. Although mortality resulting from this disease is usually lower than 1%, it can reach 20% or even higher. Bovine ephemeral fever is distributed across many countries in Asia, Australia, the Middle East, and Africa. Prevention and control of the disease mainly relies on regular vaccination. The impact of bovine ephemeral fever on the cattle industry may be underestimated, and the introduction of bovine ephemeral fever into European countries is possible, similar to the spread of bluetongue virus and Schmallenberg virus. Research on bovine ephemeral fever remains limited and priority of investigation should be given to defining the biological vectors of this disease and identifying virulence determinants.

## 1. Introduction

Bovine ephemeral fever (BEF), also known as three-day sickness or three-day fever [1], is an arthropod-borne viral disease that mainly strikes cattle and water buffalo. This disease was first recorded in the late 19th century. Historically, BEF has also been named bovine influenza, bovine epizootic fever [2], styfsieket, or dengue of cattle [3], signifying its clinical symptoms and speed of transmission.

The disease is characterized by acute high fever, anorexia, ocular and nasal discharge, excessive salivation, and muscle stiffness followed by inability to stand, reluctance to move, and a sudden drop in milk production. Mortality is usually low when diseased animals receive appropriate medical care. Direct economic losses mainly result from a significant decrease in milk production in dairy cattle and loss of condition in beef cattle. Sex predilection in BEF-affected animals is controversial. An Iranian survey demonstrated that the infection rate is significantly higher in female cattle than in males but a difference in infection rate did not exist in buffaloes [4]. On the other hand, a serological survey in 2010 on non-vaccinated cattle in Saudi Arabia gave a significantly higher seropositive rate (24.4%) in males than in females (14.6%) [5]. A survey on Tibetan yaks revealed that morbidity from BEF is higher in premature cattle and females [6].

Bovine ephemeral fever is an infectious but preventable disease. Primary vaccination in calves followed by regular boosts with quality vaccine usually provides satisfactory protection. Whether the susceptible populations are vaccinated, and which populations and individuals need to be vaccinated, depend on the BEF prevalence in the area, the value of animals, and sometimes, the risk assessment and control measures of the authorities. Vector control may be helpful, but whether it is practical and its effectiveness in BEF control may be difficult to evaluate.

In this article, recent occurrences of BEF in Asian countries are updated and summarized. Epidemiological relationships among outbreaks in different countries are discussed and some are supported by the results of phylogenetic analyses. A few directions for future BEF studies are also suggested.

## 2. Bovine Ephemeral Fever Virus and Its Susceptible Species

Bovine ephemeral fever virus (BEFV), the causative agent of BEF, is a member of the genus *Ephemerovirus* within the family *Rhabdoviridae*. The virion of BEFV is enveloped, bullet-shaped, and slightly tapered toward the rounded end. The dimensions of BEFV virion are 70 nm (ranging from 60 to 80 nm) by 145 nm (ranging from 120 to 170 nm) [7,8]. Only one serotype of BEFV has been identified so far.

Mammalian species susceptible to BEFV include domestic cattle (*Bos taurus*) and water buffalo (*Bubalus bubalis*), and most clinical BEF are observed in these two species. Seropositivity in the yak (*Bos grunniens*) [9], African buffalo (*Syncerus caffer*), waterbuck (*Kobus ellipsiprymnus*) [10], and some African wildlife [11] has also been recorded. The role of wildlife in the transmission and preservation of BEFV in nature is assumed but has not been illustrated [12]. In addition to bovine species, experimental infection of BEFV has been reported in mice, rats, guinea pigs, kittens, chicken embryos [13], and sheep [14], although these species would not be natural hosts. The results of a serological survey on pig farms in Korea led to the hypothesis that pigs could be infected with bovine arthropod-borne viruses, including BEFV, and act as a silent host of the viruses [15]. However, a follow-up study on BEFV infection in pigs to test the hypothesis remains absent.

## 3. History and Geographical Distribution

Bovine ephemeral fever is endemic in some regions of Africa, Asia, and Australia. Among Asian countries, BEF has been documented in countries from tropical to temperate areas.

### 3.1. Eastern Asia

In Asia, the earliest record of BEF may be as early as 1889 in Japan. The disease was named “bovine influenza” or “bovine epizootic fever”, characterizing the animals it affects and the clinical manifestation. During the epidemic in middle and southern Japan from 1949 to 1951, approximately 67 thousand cattle suffered from BEF, and the widespread epidemic resulted in an economic loss of two billion Japanese yen [16], equivalent to approximately 5.5 million US dollars in the 1950s. Later, in 1968, the virus was isolated from suffering cattle and was characterized serologically and biologically. This revealed that the pathogen of bovine epizootic fever is BEFV [2]. This disease in Japan has been gradually controlled since the development and use of formalin-inactivated BEF vaccine in 1966 [17]. Most BEF outbreaks in Japan after 1992 were sporadic and limited to the remote islands located at the southwestern end of Japan. The BEF outbreaks occurring in 2015 at the southern end of Kyushu, the southernmost main island of Japan, are the latest reported outbreaks recorded by the Ministry of Agriculture, Forestry and Fisheries, Japan (http://www.maff.go.jp/j/syouan/douei/kansi_densen/kansi_densen.html). The 29% mortality (24/83) in beef cattle attracted the attention of farmers and animal health agencies. [18]. This reoccurrence of BEF in Kyushu suggests that cattle on the main islands can become afflicted with BEF when their acquired immunity is insufficient.

In addition to Japan, BEF in eastern Asia has also been recorded in China [19], Korea, and Taiwan [20].

**Figure 1:**
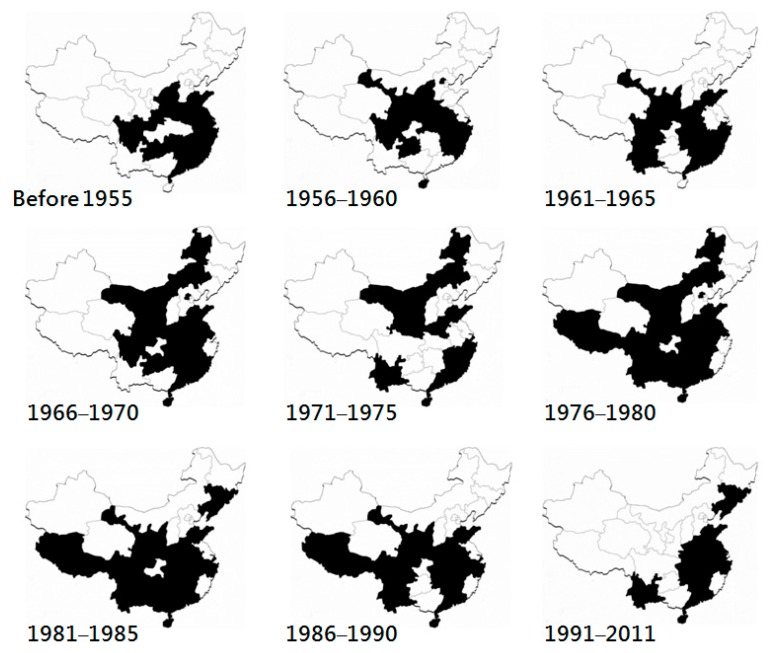
Geographical distribution of bovine ephemeral fever in mainland China from 1949 to 2011, illustrated based on the record by Zheng [21]. These maps were made on a province scale. From left-top to right-bottom, the maps demonstrate five-year periods.

Bovine ephemeral fever in China was described as early as 1934 in Jiansu Province and the first BEFV isolate in China, the JB76H strain, was obtained from diseased dairy cattle in 1976 by Lanzhou Veterinary Research Institute. Based on the epidemics documented by Zheng [21], clinical BEF has been recorded in 19 provinces, four autonomous regions (Guangxi, Neimenggu, Ningxia, and Tibet), and two municipalities (Beijing and Shanghai) of China, expanding longitudinally from 18 degrees to approximately 45 degrees north (Figure 1). The majority of the affected provinces are located in coastal and central areas, whereas the northernmost outbreaks were recorded in Hulan District (45°53′), Harbin, and Heilongjiang Province [22]. Since 1967, the disease also has appeared in Tibet, which is a plateau region in western China with an average elevation of 4500 m. The positive rates of the yak sera collected between 2012 to 2015 in Qinghai, a province north to Tibet, ranged from 28% to 40% [9]. The cattle sampled from 2014 to 2015 in Xingjian Autonomous Region gave a seropositivity rate of 90% [23]. Seropositivity in Heilongjiang, the northeastern-most province of China, was also obtained during in a nationwide survey from 2012 to 2014 [24]. These findings provide evidence that the territory of BEFV infection in China may be wider than has been clinically observed. Since the 1980s, BEF has also been reported in the northeastern provinces, Liaoning and Jilin, indicating a northward spread of the disease.

Reports of Chinese BEF outbreaks after 1990, either in international journals or to international organizations, remain absent. Most occurrences of BEF in mainland China have been recorded in domestic agriculture or livestock magazines. Information retrieved from those magazines suggests that an epidemic occurred from 2004 to 2005 in Guangdong, Hunan, Jiangsu, Shaanxi, and Zhejiang Provinces and Guizhou Autonomous Region. An epidemic in 2011 is documented as well [21].

Infection of BEFV is also present in Korea. A virological and serological survey on arthropod-borne virus infection from 2016 to 2017 demonstrated that BEFV infection was detected in counties of Jeollabuk-do, a province in southwestern Korean Peninsula [25]. Studies on the genetic characterization of Korean BEFV remain absent.

Bovine ephemeral fever first appeared in Taiwan in 1967. This disease breaks out every few years [20], and the intervals between the epidemics seem to be becoming shorter. The latest BEF outbreaks were identified in early 2014. A sequence analysis on the glycoprotein of the viruses obtained during these outbreaks indicates an invasion of an exotic BEFV strain that is genetically close to Chinese BEFV isolates [26]. No outbreak has been officially reported since the end of the outbreak of 2014.

### 3.2. South and Southeastern Asia

Information about the prevalence of BEF in southern and southeastern Asia is limited, and characteristics of BEFV isolates and their genetic analysis are unavailable. The disease has been documented in the countries including Bangladesh, Pakistan [4], Indonesia, Thailand, and the Philippines.

In Indonesia, a disease consistent clinically with BEF was recorded in Bandung, West Java, and later in Sumatran in the early 20th century. A serological survey in the early 1990s suggested that the disease was spread nationwide in Indonesia [27].

Bovine ephemeral fever in Thailand is also endemic. With a countrywide cattle population of 5.5 million heads, BEF may be one of the most important bovine diseases of the dairy and beef industry in Thailand. The first and currently only genetic study on the molecular characteristics of Thai BEFVs was published in 2018 [28]. It revealed that the BEFVs isolated from 2013 to 2017 in Thailand can be divided into two clusters. One includes the isolates obtained in China, Japan, and Taiwan from 1996 to 2014, and the other includes those obtained from China in 2011 and 2012.

The first description of bovine ephemeral fever in the Philippines was published in early 2018 [29]. Phlyogenetic analysis revealed that the Philippine BEFV is similar to the Australian BEFV isolates.

### 3.3. Middle East

Bovine ephemeral fever in the Middle East has been documented in Iran [30], Israel, Jordan, Syria, and Iraq [31], Saudi Arabia [32], and Turkey [33]. The earliest descriptions of BEF in the Middle East were in Egypt in 1924, and in the Jordan Valley and Palestine in 1931 [31]. Phylogenetical, meteorological, and chronological evidence implies that BEFV may circulate within these regions to form a single gene pool, and the introduction of an exotic strain has been suggested [34].

In Iran, BEFV was first isolated from cattle blood collected during the BEF outbreak occurring in the southern and eastern regions of Iran in 1974 [35]. Recent outbreaks were reported in September of 2006 [36] and in autumn of 2012 [30]. A virological survey carried out from August 2010 to June 2011 in Khuzestan Province of Iran showed positive rates of 29% in cattle and 17% in buffalo, suggesting that BEFV infection is prevalent at least in western Iran [4]. An outbreak in 2013 resulting in a mortality of 464 cattle was reported [37].

An outbreak occurring in Iraq from May to September of 2012 was recorded [38].

In Israel, BEF epidemics occurred in 1990, 1999, 2004, and recently, in 2008. Phylogenetical and meteorological analyses suggest that these outbreaks are likely to associate with those of Turkey [21,39].

In Saudi Arabia, the BEF situation in the country was unavailable prior to the outbreaks of 1995, although two suspected BEF outbreaks in 1980 and 1990 were reported on a basis of clinical observation [40]. The first BEF incursion occurred in 1996 in eastern Saudi Arabia, as confirmed by virus isolation and inoculation to calves. During this incursion, exotic and domestic breeds of cattle were affected [41].

The first recorded Turkish BEF outbreak occurred in 1985. Without protection with BEF vaccination, BEF has broken out in southern and southeastern Turkey every few years since 1996, that is, in 1996, 1999, 2005, 2008, and 2012. Regions of Turkey affected by the 2012 outbreak were distributed between the northern and southern borders of the country including the provinces of Southern, Eastern, and Central Anatolia, the Black Sea, and the Marmara Regions [42].

In Egypt, bovine ephemeral fever is epidemiologically associated with that in the Middle East, although Egypt geographically is not an Asian country. Egypt is one of the countries in which BEF was earliest described. The disease has persisted in Egypt for more than a century. In the 21st century, BEF broke out in Egypt in 2000, 2001, 2004, 2006, 2009, and 2010 [43,44]. Most of the affected governorates were around the Nile Delta [45].

In addition to the aforementioned countries, known epidemics in Afghanistan, Burma, and India have also been recorded [46].

## 4. Relationships between Epidemics

Bovine ephemeral fever viruses have evolved into three lineages: the lineages of Australia, East Asia, and the Middle East. Construction of the phylogeny of BEFV strains through comparing the nucleotide sequences of their viral glycoprotein gene, show a clear pattern where most of the viruses are separated with their origins of isolation [39]. This suggests that BEFVs in nature are evolving within a relatively constraint region, and the viruses in the region form a stable genetic pool. Expansion out of their existing territories occurs but is infrequent.

**Figure 2:**
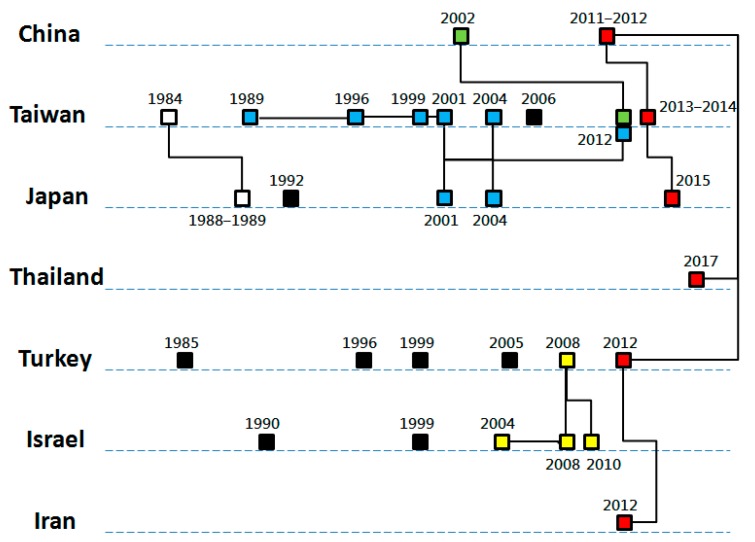
Temporal and phylogenetic relationships between bovine ephemeral fever outbreaks of Asian countries. The bricks represent the major outbreaks of the countries. The bricks filled with the same color and linked with solid lines indicate that a genetic relationship, supported by the results of phylogenetic analyses. Black solid bricks indicate outbreaks with no viral sequence available for analysis.

Phylogenetic findings support the temporal relationships between BEF outbreaks of neighboring countries. By chronological orders and geographical closeness, outbreaks that occurred in Turkey and Israel in 2004–2010 [21], in Japan in 2001–2004, in Taiwan in 1989–2012 [26], in Japan and Korea in 1988 and 1991 [47], in China in 2011–2012, in Taiwan in 2013–2014, in Japan in 2015, in Thailand in 2017, and in Turkey and Iran in 2012 [18,26] are proposed to be related (Figure 2). A surprising phylogeny was detected among the BEFVs in Iran and Turkey in 2012 and those in China in 2011, implying a long-distance BEFV dispersal [30,33].

Australia’s BEFVs are likely to circulate as an independent ecosystem to those of Asian countries. Nevertheless, based on the experiences of international transmission of bluetongue virus strains between northern Australia and southeastern Asian countries [38,48], it is possible for arthropod-borne viruses such as bovine ephemeral fever to be transmitted in the same way, although no virus isolate has shown evidence of this possibility [37].

## 5. Vectors

Competent vectors responsible for transmitting BEFV may be mosquitoes and *Culicoides*. Transmission of BEF through insect vectors has been assumed as the cause of the first occurrence of the disease in northern Australia in 1936–1937 [49]. In different continents, the virus has been isolated or its viral RNA has been detected from various species of mosquitoes and *Culicoides* biting midges, as listed in Table 1. However, the insects responsible for transmission of BEFV remain unknown.

With regard to the experimental infection in potential insect vectors, few attempts have been made. Kay et al. carried out an experiment where *Culex annulirostris* was fed BEFV-containing blood through a membrane-feeding device. It was possible to harvest the virus from this Australian mosquito after days of blood feeding [55].

## 6. Discussion

Bovine ephemeral fever is an under-rated and under-reported cattle disease. Globally, the number of cattle, including water buffalo, was estimated to be approximately one billion heads in 2018 and is increasing [56]. Of the top ten cattle rearing countries around the world, 442 million heads (44.1%) are reared in India, China, Australia, and Turkey, which are BEF endemic countries. Although direct loss resulted from mortality of BEFV-infected cattle is usually low, case fatality can be high (Table 2) when naïve areas are invaded, cattle herds with insufficient immunity are infected, or when veterinary care is not provided in time to infected herds. Moreover, considering that BEF has broken out in Turkey frequently in the past few years and some of the Turkish outbreaks have occurred geographically close to the Balkan Peninsula, sudden incursion of BEF into European countries is possible. With suitable insect vectors and appropriate climate conditions, this incursion, similar to that of the bluetongue viruses moving from northern Africa and western Asia during 1998–2005 [57], may lead to serious consequences. To improve the visibility of BEF outbreaks globally, it is encouraged that BEF should be included as one of the listed diseases by the World Organization for Animal Health. With this effort, BEF will become a notifiable disease, thus facilitating not only disease reporting but also evaluation of vaccine efficacy and the development of diagnostic methods.

Since BEF is usually recognized as a “regional” animal health problem that occurs for a lengthy period of time, few resources have been allocated to research on this disease in most countries, except perhaps in Japan and Australia. Some gaps of BEF research have been persistent, which include identification of competent insect vectors, virulence factors of BEFV, and mechanism of long-distance transmission.

Insect vectors capable of transmitting BEFV have not been identified. Colonization of the candidate vector of a given pathogen is one of the important cornerstones of studying vector competency, and it will pave the way for further research, such as pest control [59], gene expression analysis [60], and microbiota studies [61]. To the best of my knowledge, rearing methods for *C. arakawae* [62,63,64,65], *C. bambusicola* [66], *C. furens*, *C. hollensis*, *C. melleus* [67], *C. guttipennis* [68], *C. nubeculosus* [69,70], *C. peregrines* [71], and *C. sonnorensis* [72] have been documented. Nevertheless, rearing and maintaining *Culicoides* colonies in the laboratory is labor-intensive and costly, and only a few research organizations, such as The Pirbright Institute in the United Kingdom (*C. sonnorensis* and *C. nubeculosus*) and the Center for Grain and Animal Health Research in the United States (*C. sonnorensis*), are capable of stably maintaining *Culicoides* colonies. In addition to those for the American and European *Culicoides* species, techniques of rearing and colonizing *Culicoides* species abundant in Asian countries, e.g., *C. brevitarsis* and *C. oxystoma* need to be established. The establishment may not only open a window for BEF studies but also benefit the studies of other *Culicoides*-borne diseases. With regard to the vector competency in mosquitoes, although some mosquitoes are also candidates for biological vectors of BEFV and many mosquito species have been colonized in laboratories, little effort has been made to build the link between BEF studies and colonized mosquitoes of medical significance. Practically, investigating which insects are competent BEFV vectors could be much more difficult than the study of competent vectors of the bluetongue virus. As the review written by Carpenter et al [73] states, scientists went through a bumpy road, from approaching potential vectors, colonizing *Culicoides*, to establishing the link between vector-biting and infection. This process eventually broadened our understanding of vectors of the bluetongue virus. Although techniques for testing vector competence have been developed rapidly, without provision of resources and dedication to these studies, competent vectors of BEF remain a missing piece of the jigsaw.

Changes in the virulence of BEFV should be monitored. The impact of a viral infection on the host is usually associated with a number of factors, such as host immunity, predisposed diseases, and the characteristics of virus itself. In terms of the studies on the virulence of other rhabdoviruses, the variation in amino acids of the viral glycoprotein [74,75,76] shows their influences on the pathogenicity of rabies lyssavirus. In contrast, although a comparison of deduced amino acid sequences of BFV glycoprotein has been made and differences between virus isolates have been shown [21,28,33,77], influences of the amino acid variation on the pathogenicity of BEFV remain unexamined. An increase in the mortality rate was observed in the recent outbreaks in China [21] and Turkey [42], and this observation implies a change in virulence of the virus strain. A follow-up epidemiological investigation and further studies on genetic virulence determinants of BEFV are encouraged.

Wind and livestock transportation are the two major routes responsible for BEF transmission. Meteorological factors that facilitate the windborne movement of virus-infected dipterans by wind have discussed in relation to a few events: the BEF outbreak in Israel in 2004 [30], the expansion of *Culicoides imicola* into southern Europe [78,79], and the introduction of Schmallenberg virus into Ireland [80]. In addition to aerial dispersal, marine transit could be another, perhaps infrequent, latent route of transporting small insects intercontinentally. A female *Culicoides* biting midge and other insects within the family Ceratopogonidae were found in a ship traveling from Albany, Australia to Qinhuangdao, China, implying the possibility of the long-distance movement by ships [81]. With regard to livestock movement, movement either by transportation or by individual movement should be weighed up to determine their roles in BEF transmission. Livestock can move freely across borders in the Middle Eastern countries as well as across those of the countries in Indochina Peninsula. The recent introductions of Rift Valley fever into East Africa and the Middle East [82], exotic foot-and-mouth disease virus strains into southeastern Asia [83], and African swine fever virus into Vietnam in early 2019 highlight the role of livestock movement in spreading these diseases. Aziz-Boaron et al. [34] also suggested that the viruses causing BEF outbreaks in the Middle East between 2004 and 2008 were transmitted via cattle shipped from China to Jordan. Unfortunately, cross-border trades and smuggling keep increasing in some Asian regions, as exemplified by the constant livestock movement between southeast Asian countries [84] and the doubling of smuggling cases in India from 2015 to 2017 [85]. The situation may be even worse along the southern border of China. Increased protein consumption and elevating beef prices followed by booming economy of China are driving the movement of cattle without quarantine to Vietnam and China, and the risk of animal disease introduction has consequently increased.

## 7. Conclusions

Bovine ephemeral fever continues to threaten cattle populations in Asia, Australia, and the Middle East. Entomological studies on BEF insect vectors, particularly on their laboratory colonization and vector competency, as well as virological studies on virulence determinants of BEFV, are encouraged to deepen our understanding of this disease.

## Figures and Tables

**Table 1:** Detection of bovine ephemeral fever virus or viral RNA in insect vectors.

Insect	Place/Year of Sampling	Methods	References
Mosquitoes:			
*Anopleles bancroftii*	Australia/1974–1976	Virus isolation	[50]
*Culex annulirostris*	Australia/1968	Virus isolation	[51]
Biting midges:			
*Culicoides arakawae*	Korea/2016–2017	RT-PCR	[25]
*Culicoides bedfordi*	Kenya/1972–1973	Virus isolation	[52]
*Culicoides brevitarsis*	Australia/1984	Virus isolation	[51,53]
*Culicoides coarctatus*	Zimbabwe	Virus isolation	[54]
*Culicoides cornutus*	Kenya/1972–1973	Virus isolation	[52]
*Culicoides kingi*	Kenya/1972–1973	Virus isolation	[52]
*Culicoides imicola*	Kenya/1972–1973	Virus isolation	[52]
*Culicoides nivosus*	Kenya/1972–1973	Virus isolation	[52]

**Table 2:** Morbidity and case fatality of bovine ephemeral fever outbreaks or epidemics.

Year	Country	Morbidity (%)	Case Fatality (%)	References
1967	Taiwan	26.6	5.2	[58]
1983–1984	Taiwan	20.1	6.0	[58]
1989–1990	Taiwan	14.5	5.0	[58]
1990	Israel	8.3–20.0	2.0	[31]
1996	Saudi Arabia	59	>1.0	[41]
1996	Taiwan	13.6	11.3	[58]
1999	Taiwan	5.6	21.9	[58]
1999	Israel	5.4–38.7	8.6–28.0	[31]
2001	Taiwan	7.4	9.7	[58]
2001	Taiwan	15.0	50.0	[58]
2004	Israel	14.9–22.2	3.5–5.4	[31]
Before 2000	China	10.0–20.0	>1.0	[21]
2011	China	30.0	5.0	[21]
2012	Turkey	35.0	15.0–20.0	[42]
2013	Iran	17.0	25.8	[37]
2015	Japan	28.9	0.0	[18]
